# Current and Future Perspectives in Cardiac Rehabilitation

**DOI:** 10.3390/jpm12091510

**Published:** 2022-09-15

**Authors:** Shinya Tanaka

**Affiliations:** Department of Rehabilitation, Nagoya University Hospital, 65 Tsurumai-cho, Showa-ku, Nagoya 466-8560, Japan; s-tanaka@med.nagoya-u.ac.jp; Tel.: +81-52-741-2111

To reduce the morbidity and mortality of cardiac diseases, patients undergo cardiac rehabilitation consisting of a series of interventions to optimize their physical, psychological, and social functioning and to stabilize, slow, or reverse the progression of atherosclerosis underlying their condition [1]. Such rehabilitation is essential in decreasing cardiovascular disease burden, and programs for secondary prevention represent important components of preventive care. Cardiac rehabilitation has been shown to be both cost-effective and clinically effective in patients with coronary artery disease and heart failure (HF) with reduced ejection fraction [2,3,4]. The current guidelines outline the core components of cardiac rehabilitation and secondary prevention programs, and specify the means of evaluation and intervention, and the expected outcomes of such interventions [5,6,7,8].

The accumulation of further evidence is required for the application of cardiac rehabilitation in patients with other conditions, such as HF with preserved ejection fraction, atrial fibrillation, frailty, sarcopenia, and malnutrition. Outpatient cardiac rehabilitation programs have been reported to reduce the risks of all-cause mortality and HF-related hospital readmission in HF patients with preserved ejection fraction or frailty, except in cases of severe disease [9]. However, only a small proportion of patients benefit from outpatient cardiac rehabilitation, as less than 10% of institutions have implemented such interventions [10,11]. Acute HF (AHF) is often accompanied by frailty in older patients, who frequently have severe and widespread impairments to their physical function on admission to hospital [12]; moreover, hospital-acquired disability has been shown to increase the risks of both all-cause mortality and HF-related hospital readmission [13]. In frail older patients with HF, multidomain rehabilitation therapy initiated in hospital and continued on an outpatient basis following discharge was shown to lead to improvements in physical function at 3 months [14]. This large-scale trial demonstrating the effectiveness of exercise-based rehabilitation in older AHF patients with frailty has attracted a great deal of attention. Although the short-term clinical outcomes in patients hospitalized for AHF were shown to be improved with the acute-phase initiation of cardiac rehabilitation [15], frail older patients with AHF often have dyspnea, fatigue, exhaustion, and exercise intolerance, making it difficult for them to participate in exercise-based treatment programs. The multicenter, randomized, controlled ACTIVE-EMS trial, with the addition of electrical muscle stimulation (EMS) treatment to early rehabilitation, demonstrated greater improvement in lower extremity function in older patients with frailty who were hospitalized for AHF, without adverse events [16,17]. Further evidence is needed to determine the efficacy of aerobic exercise and resistance training, as well as various other types of exercise, including high-intensity interval training, inspiratory muscle training, and neuromuscular electrical stimulation, in cardiac rehabilitation.

This Special Issue welcomes submissions of original research, meta-analyses, and systematic reviews that bridge the gap between evidence and practice in cardiac rehabilitation.
